# The effects of TRX suspension training on sarcopenic biomarkers and functional abilities in elderlies with sarcopenia: a controlled clinical trial

**DOI:** 10.1186/s13102-024-00849-x

**Published:** 2024-02-26

**Authors:** Sohrab Rezaei, Rasoul Eslami, Bakhtyar Tartibian

**Affiliations:** Exercise Physiology Department, Faculty of Sport Science, Allameh Tabataba’i University, Tehran, Iran

**Keywords:** Sarcopenia, TRX suspension training, Neuromuscular factor, Growth factors, Functional abilities

## Abstract

**Background:**

Sarcopenia is an age-related progressive loss of muscle mass and strength that can be modulated by resistance training. This study aimed to investigate the effects of TRX Suspension Training (TST) on serum levels of neuromuscular and growth factors and functional indices in elderly men with sarcopenia, an age-related condition characterized by progressive muscle mass and strength loss.

**Methods:**

Nineteen sarcopenic elderly men (age = 74.87 ± 4.58 years) were randomly assigned into two groups, the TST group (*n* = 10) and the control group (*n* = 9). Serum concentrations of regulatory muscle markers, anthropometric and body composition indices, and functional tests were evaluated at baseline and after 8 weeks. The training protocol consisted of eight weeks of TRX exercises, with three weekly sessions.

**Results:**

After 8 weeks of training, growth factors such as Follistatin (FST) (*P* = 0.001), 22 kDa C-terminal agrin fragment (CAF) (*P* = 0.031), and growth differentiation factor 15 (GDF15) (*P* = 0.049) increased significantly in the training group in comparison to the control group and Myostatin (MSTN) (*P* = 0.002) had a significant decrease. However, there was no significant difference in ASMM/m2 (*P* = 0.527), SMM/m2 (*P* = 0.621), or Body fat mass (*P* = 0.433) within or between groups. In addition, the TRX Suspension Training had a significant effect on the functional tests and improved gait speed (*P* = 0.037), chair stand (*P* = 0.016), and TUG (*P* = 0.016) as well as Handgrip strength (*P* = 0.035).

**Conclusion:**

Our findings highlight the efficacy of TRX Suspension Training in enhancing the serum levels of muscle growth factors and functional capacities among elderly individuals with sarcopenia. Therefore, considering the ongoing COVID-19 pandemic, this protocol can prove beneficial for this demographic group.

**Trial registry:**

Iranian Registry of Clinical Trials identifier: IRCT20230727058944N1, prospectively registered 20-09-2023, https://en.irct.ir/trial/71635

## Introduction

Skeletal muscle is a crucial tissue in the human body, playing a vital role not only in physical performance but also in various functions of the body. To maintain this tissue considering different factors like aging, physical activity levels, and nutrition is essential [1]. Sarcopenia is an inevitable progressive loss of muscle mass and strength that is associated with aging. This situation increases the risk of impairment in physical ability which could lead to consequences such as falls, fall-related injuries, hospitalizations, and even mortality [2]. Moreover, a few articles have demonstrated a probable correlation between sarcopenia and osteopenia, often accompanied by elevated comorbidity rates in the elderly population. This evidence further emphasizes the importance of addressing sarcopenia as a critical health concern [3, 4]. The new definition of European Working Group on Sarcopenia in Older People (EWGSOP) defines sarcopenia as a muscle disease in which strength comes before mass as a parameter. Detecting low skeletal strength indicates a probable risk of sarcopenia which can be confirmed by diagnosing low skeletal muscle mass [5]. This disease is a multifactorial phenomenon and there are different stimuli that could affect skeletal muscle with aging like alterations of the neuromuscular junction (NMJ), growth factors, and inflammation [6].

Changing the levels of growth factors during the time is one of the mechanisms that could affect the sarcopenia process [7]. Myostatin (MSTN) or growth and differentiation factor 8 (GDF-8) is a member of the transforming growth factor-β (TGFβ) family and acts as a negative regulator of muscle growth [7]. MSTN mostly binds to Activin Type II receptors (ActRIIB) in muscle and by phosphorylation of Smad2/3 which blocks the AKT/mTOR signaling pathway, decreases protein synthesis, and leads to abnormal loss of muscle mass [8]. On the other hand, Follistatin (FST), acts as an antagonist of the TGFβ family [8], blocks MSTN by binding to its receptor (Activin-IIβ) or to MSTN itself, and prevents the adverse effects on muscle mass. Compared to blocking MSTN, overexpression of FST leads to more growth in muscle mass [9]. It’s been shown that the activity of FST goes beyond just blocking MSTN, it also could activate satellite cells and block Activin another negative growth factor [9].

Another member of the (TGF-β) family that is related to loss of muscle mass due to aging and sarcopenia is growth differentiation factor 15 (GDF15), also known as macrophage inhibitory cytokine 1 (MIC-1). Recently, it has been demonstrated that levels of GDF15 are associated with age and age-related diseases and disorders [10]. There is debate on the positive or negative effects of GDF15 on muscle mass. Research on mice models found that GDF15 can reduce muscle mass [11] while ablation of this factor led to increasing stress markers (Atf3, Atf6, and Xbp1s) in skeletal muscle which is unfavorable [12]. However, its role in skeletal muscle regulation could be through upregulating atrogin-1 and MuRF1 expression in muscle [13].

In addition to growth factors, it has been suggested that changes in the Neuromuscular Junction (NMJ) may have a significant impact on the development of sarcopenia [14]. NMJ is an essential component to connect nerves and muscles and is affected by age [15]. Agrin is an extracellular proteoglycan that is secreted by motor neuron axons and by binding to skeletal muscle receptors plays an important role in maintaining NMJ. An enzyme named neurotrypsin cleaves Agrin into a 22 kDa C-terminal agrin fragment (CAF) which is released into the bloodstream and can be measured in circulation [15]. Overexpression of neurotrypsin in mice models causes NMJ disorder, reduces muscle mass, and leads to symptoms of sarcopenia, also several studies showed that serum levels of CAF are much higher in sarcopenic patients [15].

On the other hand, with regard to the great benefits of exercise (especially resistance exercises) such as increasing muscle hypertrophy and improving muscle strength, performing regular resistance exercises could be a good strategy to prevent sarcopenia and its harmful effects [16]. It has been demonstrated that several motivational factors such as worrying about health, the influence of friends and family, and recommendations from a medical professional could help to increase the level of physical activity among older adults that should be noticed [17]. According to a review article [18], sedentary older adults are recommended to engage in physical activity with moderate intensity, three to five times a week. Going beyond this recommendation can potentially increase the risk of injuries and other health issues, and even diminish life expectancy [18]. As a matter of fact, sarcopenia is a complex medical syndrome in which a multidisciplinary approach should be provided. Alongside exercise, nutritional supplements could also play an important role in the treatment of sarcopenia, especially in those who suffer from the loss of muscle mass and strength of swallowing muscle (sarcopenic dysphagia) which leads to malnutrition and frailty [19]. In this study, we examined the effect of exercise on sarcopenia. Escriche-Escuder, A., et al. in a meta-analysis study showed that exercise training is beneficial for sarcopenic elderlies and improves the indicators like skeletal muscle strength and physical performance as well as a slight improvement in skeletal muscle mass [6]. For example, 8 weeks of resistance training could improve serum levels of MSTN and FST in sarcopenic men [7, 8], and a study also showed a significant decrease in CAF after 6 weeks of functional training in older adults [20].

TRX Suspension Training (TST) is one of the body weight resistance training (RT) modes that has two handles and straps in which users who are suspended from the straps use their body weight as resistance and perform multi-planar and multi-joint exercises [21]. Compared to RT, TST with upper, and lower body, and core exercises, creates an unstable environment with greater muscular demand that can put more stress on the neuromuscular system than traditional resistance exercises [22]. TST can improve muscle strength, endurance, flexibility, functional performance, and core stability, and several studies have shown a significant increase in muscle mass in older communities parallel to traditional resistance exercises [22]. On the other hand, TST can be executed as a home-based exercise training with no request for any specific gym instrumentation and associated costs. Therefore, it seems that TST could be a comprehensive exercise for elderlies and conquer the process of sarcopenia.

However, we did not find previous studies that investigated the effects of TST training on serum markers of sarcopenic people. Therefore, the limitations of previous studies on the effect of this kind of exercise on biomarkers and muscle mass improvement in sarcopenic societies and the unknown mechanisms involved in this process make future investigations necessary. Given that TST is a form of resistance training that creates an unstable environment, we hypothesized that it could activate muscle growth signaling by regulating blood growth levels. So, we investigated the effect of this kind of training on the growth and neuromuscular factors as well as the functional abilities of this group of people. Consequently, the purpose of the current study was to estimate the effects of 8 weeks of TRX Suspension Training on serum levels of sarcopenic biological markers that play a key role in sarcopenia and the functional indices in sarcopenic elders. Accordingly, the research hypothesis was that these exercises improve biological markers involved in muscle mass maintenance and functional indices in elders with sarcopenia.

## Methods

### Study design

This was a semi-experimental design (two groups in the form of a pretest and a post-test) and was conducted among the sarcopenic patient communities in Tehran, Iran. The experimental procedures and study protocols were approved by the Ethics Committee and all experimentation was carried out in accordance with the Declaration of Helsinki. In this article we used the CONSORT reporting guidelines to ensure comprehensive and transparent reporting of the study’s design and results [23]. To determine the number of participants, the G*Power analysis software was used. Volunteers were recruited from the Elderly Association of Tehran, Iran. Following that, the Handgrip test was conducted on individuals 65 or older to find those with low handgrip strength (HGS). The cut-off threshold of HGS ≤ 32 kg was utilized as a criterion to identify individuals exhibiting sarcopenia. This specific cut point was selected based on a comprehensive study conducted by Bahat G., et al. [24], which aimed to determine the appropriate cut point for the Turkish population. The rationale behind this choice stems from the similarity in genetics and physiology between the populations of Iran and Turkey. If they volunteered to participate in the program, met inclusion, and didn’t have the exclusions, they were eligible to join the study.

The inclusion criteria were as follows: (1) age more than 65 years (their age was verified by their identification number); (2) handgrip strength lower than 32Kg (it was assessed with a dynamometer) [24], SMM/height^2^ lower than 9.2 kg/m^2^ (assessed by body composition) [24]; (3) being sedentary for at least 1 year (didn’t have more than 1 h exercise per week) [16]. Exclusion criteria included (1) cardiovascular or pulmonary diseases, diabetes, Joint and muscle problems, and Mental and cognitive disorders (they were asked if they have any health problem and if they have any medical record related to our exclusion) [16]; (2) involvement in any extra exercise training programs; (3) not interested to continue or change in personal life schedule. The participants were also asked not to abuse any drugs or alcohol. The participants who met the inclusion criteria were randomly assigned into two groups: control (*n* = 9) and TST (*n* = 10) groups. The initial demographic characteristics of participants for different groups are shown in Table 1.

**Table 1:** Demographic characteristics of subjects with different groups in initial state

	Control (*n* = 9) (mean ± SD)	TST (*n* = 10) (mean ± SD)
Age (years)	76.5 ± 3.53	72.5 ± 4.17
Weight (kg)	63.98 ± 5.84	69.7 ± 2.35
BMI (kg/m^2^)	23.2 ± 2.80	23.45 ± 0.90
Body Fat (%)	24.40 ± 5.12	21.28 ± 3.88
Handgrip strength (kg)	27.25 ± 1.39	31.00 ± 0.76
SMM (kg)	23.59 ± 1.74	26.21 ± 1.09
SMM/height^2^ (kg/m^2^)	8.58 ± 0.54	8.72 ± 0.55
ASMM/height^2^ (kg/m^2^)	6.27 ± 0.46	6.46 ± 0.39

TST: TRX suspension Training, ASMM: Appendicular skeletal muscle mass, SMM: Skeletal muscle mass, BMI: Body mass index

**Fig. 1:**
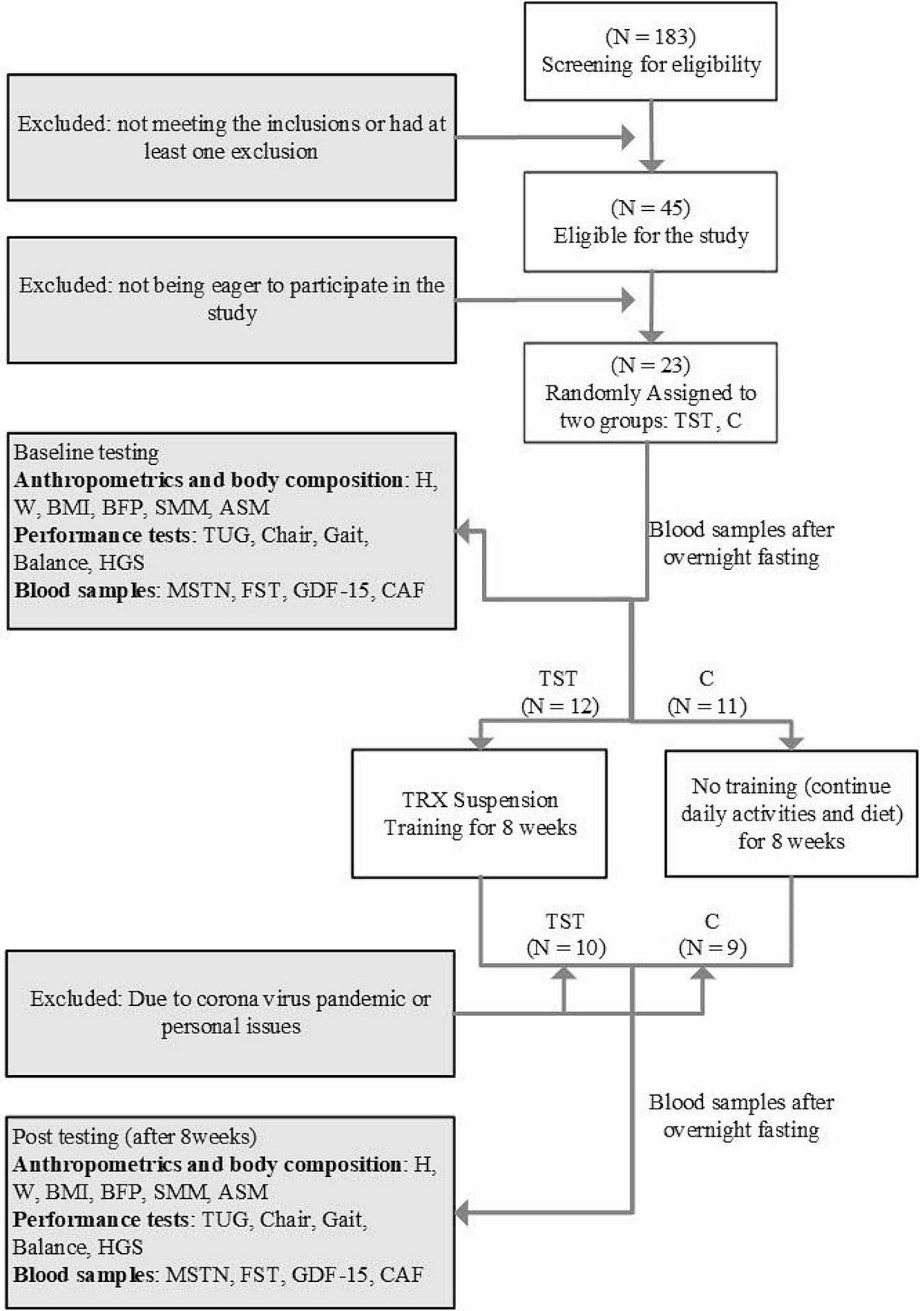
A schematic of the study design. Anthropometric, Body composition, blood tests were performed before and after of the intervention with 8 h of fasting at 9–10 AM. Abbreviations: RT: resistance training, C: control, H: height, W: weight, BMI: Body mass index, BFP: Body fat percentage, SMM, skeletal muscle mass, ASMM: appendicular skeletal muscle mass, WHR: waist to hip ratio, CC: calf circumference, HGS: handgrip strength, TUG: Timed-up-and-go test, MSTN: Myostatin, FST: Follistatin, GDF-15: growth differentiation factor 15, CAF: C-terminal agrin fragment

Participants in the Control group were asked to continue their routine lifestyle and instructed to keep a diary regarding exercise during the experiment period. The experimental group executed the TRX Suspension Training. The main protocol of this study lasted for 4 months: 1 month of sarcopenia evaluation during which suitable individuals meeting the criteria of the study were identified, 2 months of training comprising three sessions per week, each lasting one hour, and 1 month of post-evaluation, involving the measurement of anthropometric indices, collection of blood samples, and laboratory assessments. In addition, 24 h before and 48 h after the training protocol, body composition, body mass index (BMI), Functional tests, and blood sampling (after overnight fasting) were executed for two days in a gym near the training place. Participants were asked not to change their lifestyle and dietary habits during the study. A schematic of the study design is presented in Fig. 1.

### Anthropometric indices and body composition

Standing Height and Waist, hip, and calf circumference were measured with measuring tape. An Inbody770 machine (In body; Model: Zuse 9.9, South Korea) was used to measure weight, BMI, Body fat percentage (BFP), skeletal muscle mass (SMM), and appendicular skeletal muscle mass (ASMM). Before every measurement, we cleaned all 8 electrodes of the alcoholic pad to minimize the contact noise. Due to the sensitivity of BIA to hydration, participants were asked not to use alcohol and not to do vigorous exercises 24 h before the measurement. To reach this purpose, the body compositions of participants were also measured between 8 a.m. to 9 a.m. after at least 8 h of overnight fasting with an empty bladder [25]. Inbody770 results have a strong correlation with DXA and to improve the accuracy of BIA, a formula was used to measure the exact SMM:

SMM = 4.01 + 0.28 * BMI + (-2.93) * Gender + 0.61 * SMM-by-BIA + 0.001 * (SMM-by-BIA)^2^ + 0.10 * fat percent-by-BIA [25].

In addition, another formula was used to measure the exact ASMM:

ASMM = 5.07 + 0.26 * BMI + (-1.19) * Gender + 0.24 * ASMM-by-BIA + 0.01 * (ASMM-by-BIA)^2^ + (-0.06) * fat percent-by-BIA [25].

### Handgrip strength

For the handgrip test, we used The American Society of Hand Therapists (ASHT) recommendation for standard positioning. Subjects were in a sitting position with a straight back, shoulders adducted and neutrally rotated, the elbow was flexed to 90^o^, the forearm was in a neutral position and the wrist was between 0 and 30° of dorsiflexion. Jamar hand dynamometer (USA) which has five handle positions was used in this study, and a second position was used for all participants [26]. The test was executed three times with a 60 s rest period in between and the best result was recorded in kilograms (kg).

### Blood sampling and analysis

One day before starting training protocols and 48 h after the last training sessions, blood samples (5 ml) were collected from the cubital veins at the same time in the morning and 12 h fasting state. Vacutainer tubes containing a clot activator and serum gel separator were used to obtain the blood samples. Samples were placed at laboratory temperature for 5 min to complete the clotting reaction. Then they were centrifuged (10 min, 3000×g) and kept in a cryovial tube at a temperature of -20 ^o^C. Enzyme-linked immunosorbent assay method was used to measure serum levels of Myostatin, Follistatin, GDF-15, and CAF. All factors were assessed by ELISA using the ZellBio Germany kit with the following catalog numbers: MSTN: (Cat No: ZB-0403-H9648), FST: (Cat No: ZB-11,010 S-H9648), GDF-15: (Cat No: ZB-11,753 S-H9648), CAF: (Cat No: ZB-13,971 S-H9648).

### Functional tests

To evaluate the physical performance of the subjects, the following functional tests were used.

#### Gait speed

Two lines were marked on the floor with a distance of 3 m. Participants should stand behind the first line and they were asked to walk toward the second line at normal speed, just as they were walking in the street or going shopping. Subjects were asked to start the walk with a command of “go”. As they started walking, time was started with a stopwatch and stopped when the participant’s feet were completely across the end line. Time was recorded in seconds with an accuracy of 0.01 s [27].

#### Time up and go (TUG)

Subjects started the test from a chair while sitting on a chair. A line was marked on the floor at a distance of 3 m from the chair. The procedure was completely described for the participants. Time started with the command of “go” and the subject should stand up from the chair and start walking towards the line and when they arrived at the line they should turn back and sit on the chair again. As the participant lay his back on the chair again the time stopped and recorded in seconds with an accuracy of 0.01 s [27].

#### Chair stand

The test started in a sitting position and subjects were asked to get up from the chair 5 times, as fast as they could and without stopping, with their arms crossed on their chests. Subjects should stand up fully each time they stand and sit down completely while sitting. The test time started with the “go” command and stopped when the subject sat for the fifth time. Test time or inability to complete the test was recorded [27, 28]..

#### Standing balance test

This test consists of 3 steps. In the first stage (Side-by-Side Stand), the subject should stand with his feet together and must maintain balance for 10 s. Bending the knees, and shaking the arms and body was allowed, but the legs should not move. If the subject succeeded in standing in this position for 10 s, he could go to the next step and if not, the time was recorded. In the second stage (Semi-Tandem Stand), the subject should stand in such a way that the side of the heel of one foot should be in contact with the big toe of the other. The subject can put his left or right foot (whichever is more comfortable) forward. The situation was the same as in the first stage, and if the subject maintains his balance for 10 s, he goes to the next stage. In the third stage (Tandem Stand), one foot (whichever is more comfortable) should be placed exactly in front of the other foot. The conditions are the same as the previous steps and the subject time is recorded. For scoring, if a person cannot finish the first stage, he will not get any points, if the first stage was completed, 1 point, if the second stage is completed, 2 points, if the third stage is completed in three seconds to nine seconds, 3 points, and if the subject completes all three stages, he would get 4 points [28].

### TRX suspension training procedure

The training programs included 8 weeks, three sessions per week, about 60 minuntes per session including warm up and cool down. Every training session was divided into three parts: (A) warming up (10 min), (B) main training (according to weekly duration) (40 min), and (C) cooling down (10 min). The main body training consisted of 6 exercises (two exercises for the upper body (Push-up and Rowing), two exercises for the lower body (Squat and Lateral Lung), one exercise for the abdominal muscles (Lateral Flexion), and one exercise that involves the whole ventral muscle chain (Upper Body Extension) (Table 2). Each exercise consisted of 3 sets of 12 repetitions with 1 min of rest between each set. According to the previous study [21], to obtain 1RM of each participant, a marked mat was placed next to the anchor and each mark on the mat indicated the difficult stages of the exercise. On the day of the introduction of the exercises, the participants were asked to do the exercises, and a specific location on the mat where they could not do more than 15 to 20 repetitions of each exercise was recorded in a notebook. According to the 1RM-repetition number relationship, being able to do an exercise with around 15 to 20 repetitions, the exercise is performed with an intensity of approximately 60–65% 1RM. Also, this process was repeated every 2 weeks, and training intensity increased according to the individual’s progress. The intensity of training increased according to two principles, the principle of body part contact and then the principle of body angle. To increase the intensity, if the participant was doing the exercise with legs a little wider than shoulder-width apart, he was asked if he could do 15 to 20 repetitions with his feet together, and if he was doing the exercise with his feet together, the principle of body angle was used to increase the intensity and he was asked if he could do 15 to 20 repetitions on the harder level on the marked mat with his legs wide apart. In all exercises, participants must hold the TRX handles firmly. In the cooling section, the participants performed stretching exercises for 10 min.

**Table 2:** TRX suspension training protocol

Exercise Type		Number of sets	Number of Repetition	Resting time	sling length	mat position				Exercise progression
Squat		3	10–15	60s	short	-	-	-	-	Increase the repetition base on the progression of subjects
Lateral Lunge		3	8–12	60s	short	-	-	-	-	
Push-Ups		3	12	60s	short	4 Hard	3	2AP	1 Easy	Exercise progression Increased every 2 weeks base on the individual’s progress, according to two principles: 1- principle of body part contact and 2- principle of body angle
Rowing		3	12	60s	short	4 Easy	3	2	1AP Hard	
Body Extension		3	12	60s	short	4AP Hard	3	2	1 Easy	
Lateral Flexion		3	12	60s	short	4 Easy	3	2	1AP Hard	

The exercise intensity was increased moving from easy to hard points on the mat. For squat and lateral raise, the intensity was increased by increasing the repetition of exercises

AP: Anchor Point

### Statistical methods

The number of participants was estimated by G*Power analysis software (version 3.1). Based on α = 0.05, and a power (1 − β) of 0.80, the sample size needed for this project to detect significant changes in the blood factors between groups was at least 24 participants (*n* = 12 for each group). The normality of data was assessed using the Shapiro-Wilk test. To examine between‑group differences following the 8‑week training program, the Repeated Measure ANOVA test was used. Paired t‑tests were employed to compare the changes in each group (pretest to post-test). All analyses were performed using SPSS (version 21). Statistical significance was accepted if *p* ≤ 0.05.

## Results

### Participants

A month prior to the program’s initiation, the eligibility of a total of 183 elderly individuals was assessed. According to the study criteria for participant selection, 45 non-athlete sarcopenia elders were selected. Prior to participation, informed consent was obtained from all participants. However, as a result of the ongoing coronavirus pandemic, a total of 26 participants expressed a change of heart regarding their participation in the study and subsequently withdrew before its commencement (Fig. 1). Furthermore, as a consequence of the pandemic, some participants declined to participate in the training protocol. Therefore, they were all asked about their willingness to participate in the training program, and those who expressed a preference for participation were assigned to this group. In order to address the consequent reduction in sample size, the approach of duplicate laboratory testing was employed to thoroughly examine the blood samples.

### Body composition indexes

Body composition indices did not have significant changes neither between nor within groups. Data analysis revealed that main effect for Time of weight [F = 0.501, *p* = 0.506, *η*^2^ = 0.077], BMI [F = 3.774, *p* = 0.100, *η*^2^ = 0.386], WHR [F = 0.131, *p* = 0.730, *η*^2^ = 0.021], calf circumference [F = 2.160, *p* = 0.192, *η*^2^ = 0.265], Body fat percentage [F = 4.095, *p* = 0.089, *η*^2^ = 0.406] and Appendicular muscle mass/m^2^ [F = 0.664, *p* = 0.4496, *η*^2^ = 0.100] was not significant. These results represent that the body composition indices were not significantly different between pre and post-time for groups (Table 3).

Data analysis also showed that Time x group interaction effect of weight [F = 0.682, *p* = 0.440, *η*^2^ = 0.102], BMI [F = 0.847, *p* = 0.393, *η*^2^ = 0.124], WHR [F = 0.125, *p* = 0.736, *η*^2^ = 0.020], calf circumference [F = 2.160, *p* = 0.192, *η*^2^ = 0.265], Body fat percentage [F = 0.707, *p* = 0.433, *η*^2^ = 0.105] and Appendicular muscle mass/m^2^ [F = 0.451, *p* = 0.527, *η*^2^ = 0.070] was not significant (Table 3). This means that after the eight weeks of training body composition indices were not significantly different than before training.

Also, no significant main effect for group was obtained for weight [F = 2.855, *p* = 0.142, *η*^2^ = 0.322], BMI [F = 0.847, *p* = 0.393, *η*^2^ = 0.124], WHR [F = 0.125, *p* = 0.736, *η*^2^ = 0.020], Body fat percentage [F = 0.739, *p* = 0.423, *η*^2^ = 0.110], Appendicular muscle mass/m^2^ [F = 0.515, *p* = 0.500, *η*^2^ = 0.079] and calf circumference [F = 4.523, *p* = 0.078, *η*^2^ = 0.430] (Table 3). Thus, there were no overall differences between the training and control groups except for Appendicular muscle mass/m^2^ and calf circumference in which there was a significant difference between groups.

### Functional tests

Repeated measures ANOVA for the Handgrip test [F = 3.000, *p* = 0.134, η2 = 0.333], Chair stand test [F = 2.534, *p* = 0.163, η2 = 0.297], and Gait speed [F = 4.761, *p* = 0.072, η2 = 0.442] showed no significant main effect for Time, however for TUG test, the main effect for Time was significant [F = 10.087, *p* = 0.019, η2 = 0.627]. This means that no difference has been seen between the pre and post-time of the two groups except for the TUG test in which the pairwise comparison test showed that in the training group, the post-test of TUG was significantly lower than the pre-test (*p* < 0.05).

A 2 (Time) × 2 (Group) mixed-model ANOVA for Chair stand test [F = 1.267, *p* = 0.303, η2 = 0.174], and TUG test [F = 2.782, *p* = 0.146, η2 = 0.317] demonstrated that the main effect for the group was not significant that shows no significant difference between the training and control groups. These results are in contrast to the main effect of Time for Handgrip test [F = 41.361, *p* = 0.001, η2 = 0.873], and Gait speed [F = 11.534, *p* = 0.015, η2 = 0.658] that demonstrate the difference between groups was significant.

The results of Handgrip test [F = 7.408, *p* = 0.035, η2 = 0.553], chair stand test [F = 10.884, *p* = 0.016, η2 = 0.645], TUG test [F = 10.987, *p* = 0.016, η2 = 0.647], and Gait speed [F = 7.182, *p* = 0.037, η2 = 0.545] showed significant difference in Time x group interaction effect. These results indicate significant improvement in all functional tests of the training group in comparison to the control group.

**Table 3:** Serum factors, muscle mass and strength, and functional tests results

Variables	Variables	Control (*n* = 9) (mean ± SD) Paired comparison (*P*-value)	TST (*n* = 10) (mean ± SD) Paired comparison (*P*-value)	Time effects *p*-value	Interaction effect (time*group) *P*-value
Weight (Kg)	Pre:	63.98 ± 5.84	69.70 ± 2.35	*P* = 0.151	*P* = 0.440
	Post:	63.95 ± 6.68	70.03 ± 2.80	Eta-squared = 0.311	Eta-squared = 0.102
		*P* = 0.996	*P* = 0.666		
BMI (Kg/m2)	Pre:	23.20 ± 2.80	23.45 ± 0.90	*P* = 0.292	*P* = 0.439
	Post:	23.38 ± 2.91	24.57 ± 0.98	Eta-squared = 0.137	Eta-squared = 0.077
		*P* = 0.921	*P* = 0.837		
Myostatin (ng/L)	Pre:	57.40 ± 9.55	67.31 ± 11.46	*P* = 0.002	*P* = 0.002
	Post:	57.36 ± 10.31	60.26 ± 6.92	Eta-squared = 0.307	Eta-squared = 0.303
		*P* = 0.983	*P* < 0.001		
Follistatin (ng/ml)	Pre:	5.43 ± 1.28	5.70 ± 1.29	*P* = 0.004	*P* = 0.001
	Post:	5.38 ± 1.06	6.24 ± 1.33	Eta-squared = 0.272	Eta-squared = 0.356
		*P* = 0.689	*P* < 0.001		
GDF15 (ng/L)	Pre:	290.34 ± 36.38	340.12 ± 66.92	*P* < 0.001	*P* = 0.049
	Post:	300.21 ± 39.91	365.68 ± 75.04	Eta-squared = 0.381	Eta-squared = 0.141
		*P* = 0.035	*P* = 0.001		
CAF(pmol/L)	Pre:	2.66 ± 0.52	3.57 ± 0.84	*P* = 0.003	*P* = 0.031
	Post:	2.74 ± 0.46	4.06 ± 0.96	Eta-squared = 0.289	Eta-squared = 0.168
		*P* = 0.540	*P* = 0.001		
HGS (Kg)	Pre:	27.25 ± 1.5	31.75 ± 0.5	*P* = 0.134	*P* = 0.035
	Post:	26.25 ± 0.96	36.25 ± 3.86	Eta-squared = 0.333	Eta-squared = 0.553
		*P* = 0.308	*P* = 0.093		
Chiar test [29]	Pre:	10.53 ± 2.19	9.57 ± 1.60	*P* = 0.163	*P* = 0.016
	Post:	10.81 ± 2.00	8.78 ± 1.74	Eta-squared = 0.297	Eta-squared = 0.645
		*P* = 0.135	*P* = 0.074		
TUG [29])	Pre:	8.35 ± 1.04	8.44 ± 0.47	*P* = 0.019	*P* = 0.016
	Post:	8.40 ± 0.93	6.22 ± 1.38	Eta-squared = 0.627	Eta-squared = 0.647
		*P* = 0.651	*P* = 0.047		
Gait speed [29]	Pre:	2.45 ± 0.45	1.80 ± 0.14	*P* = 0.072	*P* = 0.037
	Post:	2.47 ± 0.46	1.54 ± 0.13	Eta-squared:0.442	Eta-squared = 0.545
		*P* = 0.558	*P* = 0.079		
SMM/h2(Kg/m2)	Pre:	8.58 ± 0.54	8.72 ± 0.55	*P* = 0.336	*P* = 0.621
	Post:	8.57 ± 0.52	8.67 ± 0.46	Eta-squared:0.154	Eta-squared = 0.043
		*P* = 0.963	*P* = 0.885		
ASMM/h2(Kg/m2)	Pre:	6.27 ± 0.50	6.46 ± 0.42	*P* = 0.446	*P* = 0.527
	Post:	6.21 ± 0.48	6.46 ± 0.37	Eta-squared:0.100	Eta-squared = 0.070
		*P* = 0.200	*P* = 0.941		
Body fat(%)	Pre:	24.40 ± 5.12	21.28 ± 3.88	*P* = 0.423	*P* = 0.433
	Post:	24.87 ± 5.54	22.43 ± 3.67	Eta-squared:0.110	Eta-squared = 0.105
		*P* = 0.911	*P* = 0.245		

Values are expressed as mean ± SD and (95% CI). Data were assessed by paired t-test (differences between PRE and POST within groups) and Repeated measures ANOVA (differences between groups). Level of significance was set at 0.05

TST: TRX Suspension Training, GDF-15: growth differentiation factor 15, CAF: C-terminal agrin, HGS: handgrip strength, TUG: Timed-up-and-go test, ASMM/h^2^: Appendicular skeletal muscle mass/height^2^

### Serum factors

**Fig. 2:**
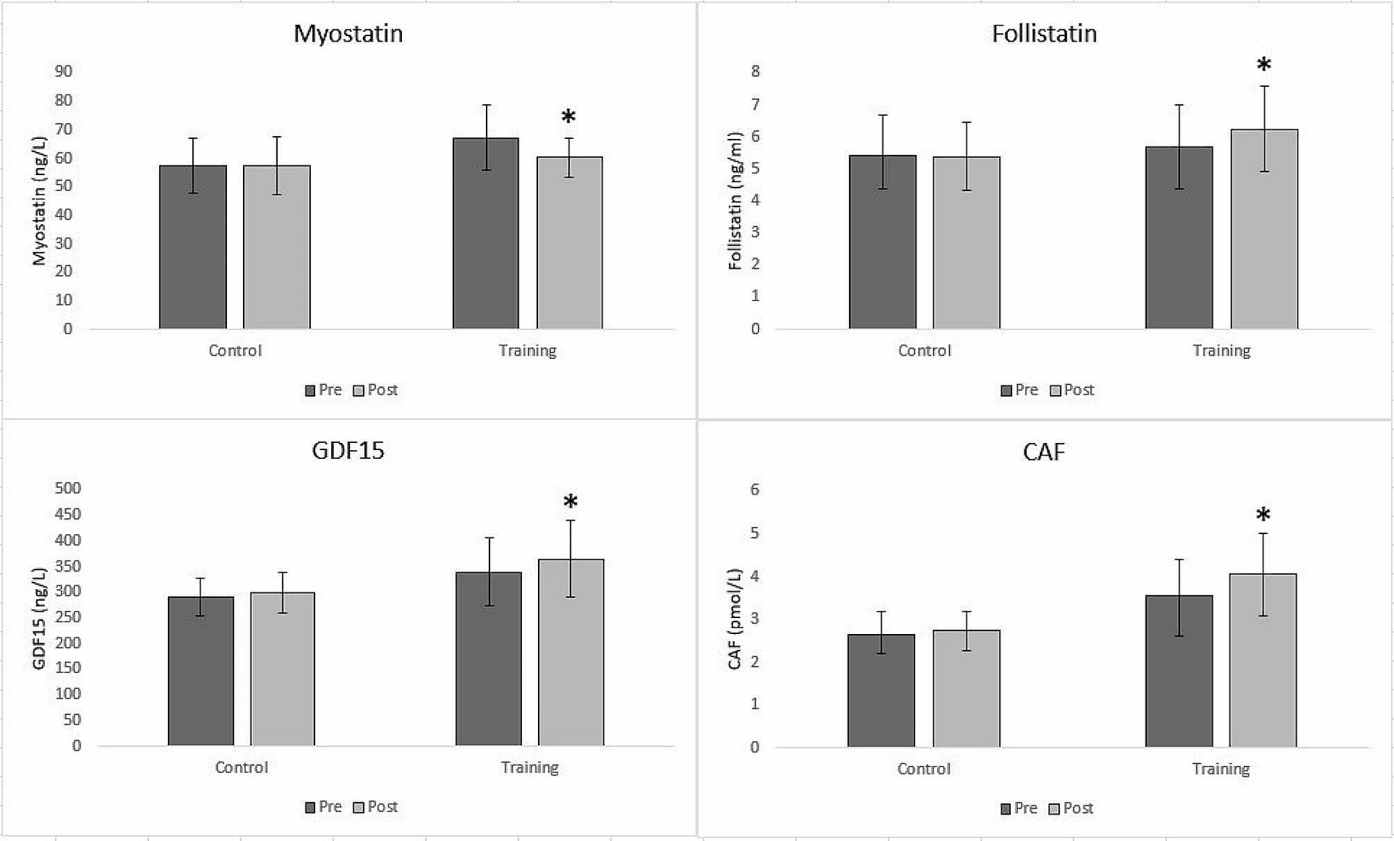
Myostatin, Follistatin, GDF-15 and CAF contents in two groups (TRX Training and Control) in pre and post-training *: a significant difference in the training group in comparison to the control group

For serum factors, a 2 (Time) × 2 (Group) mixed-model ANOVA for MSTN [F = 11.494, *p* = 0.002, η2 = 0.307], FST [F = 9.727, *p* = 0.004, η2 = 0.272], GDF15 [F = 23.783, *p* < 0.001, η2 = 0.435], and CAF [F = 10.588, *p* = 0.003, η2 = 0.289] demonstrated that the main effect for Time had strong significance (Fig. 2). Pairwise comparison test revealed that for MSTN only in training group we had a significant decrease (*p* < 0.001) between the pre and post-time, and for FST and CAF post-test results were significantly higher than pre-test (*p* < 0.001). However, the pairwise comparison test showed that in both the training group (*p* < 0.001) and the control group (*p* = 0.035), the post-test of GDF15 was significantly higher than the pre-test.

Data analysis for GDF15 [F = 6.610, *p* = 0.016, η2 = 0.203] and CAF [F = 17.012, *p* < 0.001, η2 = 0.385] showed a significant main effect for group, however, it was not significant for MSTN [F = 3.268, *p* = 0.082, η2 = 0.112] and FST [F = 1.364, *p* = 0.253, η2 = 0.050]. Therefore, in contrast to MSTN and FST, there were significant differences in the training group for GDF15 and CAF in comparison to the control group.

The results of all factors (MSTN [F = 11.279, *p* = 0.002, η2 = 0.303], FST [F = 14.388, *p* = 0.001, η2 = 0.356], GDF15 [F = 4.500, *p* = 0.044, η2 = 0.148], and CAF [F = 5.232, *p* = 0.031, η2 = 0.168]) were significant for Time x group interaction effect. Thus, there were significant increases in the training group for FST, GDF15, and CAF and a significant decrease for MSTN, in comparison to the control group.

## Discussion

To the best of our knowledge, this is the first study that examined the effects of TST on both functional performance and sarcopenia-related biological markers in sarcopenic men elderly. Our hypotheses were confirmed: It was found that 8 weeks of TST led to improving functional and biological markers involved in sarcopenia. After 8 weeks of TST, serum levels of Myostatin were significantly decreased and Follistatin, GDF-15, and CAF were significantly increased. However, it couldn’t significantly change ASMM and body composition in older men with sarcopenia. In addition, 8 weeks of TST improved the physical performance of the subjects as functional tests (Chair stand, TUG, and Gait speed) and handgrip strength.

In line with this, several studies have been conducted about growth factors improvement by exercise training, especially for MSTN and FST and most of them reached the same results as in the current study. For example, an investigation by Mafi, F., et al. [8] demonstrated that after 8 weeks of whole-body resistance training with an intensity of 60–80% of 1 RM on sarcopenic males, MSTN decreased significantly. The same results were observed in another study, where a significant decrease in serum MSTN concentrations was reported after 8 weeks of progressive resistance training on Male sarcopenic subjects [7]. It also has been shown that after a single bout of resistance exercise, the Serum level of MSTN was significantly decreased immediately and 4 h after exercise [30]. Furthermore, a comprehensive meta-analysis study has revealed that resistance training has the capability to effectively diminish Myostatin levels in adult individuals [31]. While the short-time effect of resistance training on MSTN shows significant improvement, it seems that in the long term, this effect is reduced. In this regard, MSTN levels in older adults participating in a 20-week high-velocity resistance training program didn’t change significantly [32]. Another study reported growth in MSTN gene expression after 21 weeks of progressive resistance training in older adults compared to young men [33]. MSTN binds to one of the two activin type II receptors (mostly ActRIIB), leading to the phosphorylation and activation of Smad2 and Smad3. This myostatin–SMAD pathway Blocks AKT which suppresses protein synthesis through the AKT/mTOR pathway and increases protein degeneration by enhancing the expression of MAFbx and MuRF1 through the AKT-FOXO pathway [34]. So, the reduction of MSTN could enhance protein synthesis, stop muscle degeneration, cease losing muscle mass during old age, and maybe reverse the sarcopenia process‌. In our study, 8 weeks of TST could reduce MSTN which may help to reverse muscle atrophy pathways and protein synthesis suppression.

Follistatin (FST) is another growth factor that plays an important role in the regulation of muscle mass. Several studies demonstrate significant increases in FST after resistance training [8]. Negaresh, R., et al. [7] reported that after 8 weeks of resistance training, FST increased significantly in sarcopenic older adults. In this regard [35], showed that after 8 weeks of resistance training on young and old men, both groups experienced significant growth in FST. It also has been shown that 8 weeks of concurrent training could raise FST and reduce MSTN significantly in sarcopenic elderly [16]. A single bout of resistance training in elderly men [30] and 24 weeks of elastic band resistance training in elderly women [36] also had a significant effect on FST and increased it. These results illustrate that resistance training would mostly have a positive effect on FST. In line with the above data, our results showed that following 8 weeks of TST, the FST level increased. The FST levels affect protein synthesis as well as muscle mass through different pathways. It binds to activin type II receptors and even MSTN, therefore it restricts the activity of MSTN and other members of the TGFβ family like Activin, and overexpression of FST could activate satellite cells and probably increase protein synthesis [9]. Another possible effect of FST overexpression on muscle is by improving insulin resistance which reinforces insulin’s action and increases insulin-stimulated intracellular insulin signaling of AKT/mTOR that increases protein synthesis and up-regulates muscle mass [37]. Enhancing the production of proteins and preventing their breakdown promotes the preservation of muscle mass and enhances muscle function in sarcopenia [38]. These data demonstrate that increasing FST levels leads to protein synthesis and muscle growth which could stop the process of sarcopenia and even improve muscle mass in sarcopenic elderly.

In our research, it also has been shown that eight weeks of TST could increase GDF-15 significantly in the male sarcopenic community. However, levels of this factor also experienced a significant increase in the control group. A limited number of studies in this area showed that 12 weeks of aerobic training at ∼ 85% maximum heart rate in older adults with obesity led to a significant increase in GDF-15 [39]. Nevertheless, the study administered by Hofmann, M., et al. [36] showed that after 12 and 24 weeks of elastic band resistance training, GDF-15 levels had no significant changes in elderly women. The same result has been demonstrated by [10] in which eight weeks of resistance training with 70% 1RM in elderly men could not change GDF-15 significantly.

This difference may be attributed to the fact that there is a controversy over the beneficial or detrimental effects of GDF-15 on body and muscle mass. In regards to its effect on muscle, it has been shown that elevated levels of GDF-15 caused quadriceps atrophy in patients who had cardiac surgery [40]. In addition, in patients with cancer, reduction of GDF-15 through inhibiting of its receptor GFRAL, led to a treatment for cancer cachexia decreasing several atrophy-related genes such as atrogen1, Gadd45α, and Bnip3 [41]. Moreover, overexpression of GDF15 in muscle could lead to muscle atrophy by activating FOXO1 and SMAD3 but it is not clear from which receptor GDF-15 activates this pathway [42]. On the other hand, in experimental models, GDF-15 attenuates cardiac hypertrophy through the SMAD pathway [43] and plays a protective role for heart and endothelial cells [44]. In patients with cancer, while the majority of studies showed that GDF-15 could enhance cancer cell apoptosis, some of them indicated the adverse effects of this factor [45]. In addition, physiological conditions like exercise induce GDF-15 could have a protective role against obesity and insulin resistance [42] and also eliminate its reinforced stress markers like Atf3, Atf6, and Xbp1s in skeletal muscle after exercise [12]. Considering the mentioned information, it seems that GDF-15 acts differently in various conditions. Most studies have seen GDF-15 as a protective and beneficial factor in different tissues, however in skeletal muscle, although, it attenuates stress markers, it seems that it may activate atrophy pathways too. In conclusion, as mentioned earlier, GDF-15 could have an adverse effect on sarcopenia and decrease skeletal muscle mass by activating FOXO1 and SMAD3, however, its protecting role against insulin resistance could reverse the process of sarcopenia. Regarding these findings, more studies are needed to investigate the effect of this factor on skeletal muscle mass and sarcopenia.

Another mechanism that is considered to have a role in the progression of sarcopenia is denervation. NMJ integration is necessary to maintain motor nerves and muscle fibers and its deficiency is linked to sarcopenia progression [15]. Agrin plays an important role in mediating pre-and post-synaptic communication and crosstalk between nerve and muscle through Agrin-Musk-Lrp4 Signaling Cascade results in the acetylcholine receptors (AChRs) clustering which is the main event in the differentiation of NMJs [15]. As previously mentioned, in the NMJ remodeling process, agrin is cleaved by an enzyme named neurotrypsin, into a 22 kDa C-terminal agrin fragment (CAF) which is measurable in the bloodstream. CAF is suggested as a biomarker of sarcopenia because of its inverse relationship with NMJ stability [15], muscle mass [14], and also its elevated levels in sarcopenic elderly compared to the aged healthy subjects, thus CAF is assumed as a biomarker of NMJ disruption and sarcopenia [20]. Recent studies showed that exercise training upregulates the expression of agrin and decreases neurotrypsin in NMJ as well [46]. Following these data and the fact that neuromuscular adaptation happens in response to physical activity, especially in TST [21], we expect a decline in CAF levels in the blood. In contrast, in our study, after 8 weeks of TST, serum levels of CAF had a significant increase in the sarcopenic elderly. In this regard [47], reported the same result, as, after 6 weeks of resistance training CAF circulating levels increase significantly in older adults. However, 12 weeks of power and strength training with consumption of vitamin D3 [14], and 6 weeks of functional training in older adults [20], demonstrated a significant decrease in CAF levels. In another study, one year of physical activity program did not have a significant impact on serum CAF concentrations [48]. Pratt J., et al. [15] suggested that these changes following the exercise represent the advantage of this method to subside NMJ instability. According to a review article, power and resistance training have been found to be more effective than aerobic exercises in reducing CAF [49]. However, it is important to note that most of the studies included only elderly subjects, and the prevalence of sarcopenia was not assessed in these individuals. It is possible that training in sarcopenic elderly individuals may yield different effects compared to those without sarcopenia. Additionally, other factors like different comorbidities could be effective on CAF levels, highlighting the necessity for comprehensive research to understand the reasons behind changes in CAF levels in different situations [50]. Despite the positive effect of exercise training on NMJ, it seems that further investigations are needed to describe this difference in the effect of exercise training on levels of CAF concentration.

Based on our functional performance tests, our results indicated significant improvement in muscle strength (HGS), and physical performance (chair stand, TUG, gait speed), suggesting a neuromuscular adaptation following TST. However, body composition (fat percent, muscle mass, and ASMM) did not show any significant change. According to the latest definition of the EWGSOP, muscle strength is prior to muscle quantity in diagnosing sarcopenia and its difficulties. As low muscle strength is confirmed, sarcopenia is probable and with the detection of low muscle mass, sarcopenia is approved. In addition, the level of physical performance can predict the severity of sarcopenia [5]. In line with this, it has been shown that resistance training is a proper activity to prevent sarcopenia and its harmful consequences. A meta-analysis [6] reported the same results and mentioned that although resistance training had positive effects on muscle strength and physical performance, it does not have a significant effect on muscle mass in sarcopenic elderlies. Therefore, we can establish that 8 weeks of TST has the potential to improve strength without muscle mass growth. This result was predictable because TST is a type of functional training in which neuronal adaptations are more dominant over the muscle itself. However, it seems that using protein supplements alongside exercise training helps the process of hypertrophy and leads to growth in muscle mass in this community [51].

Functional performance and self-independency have a more important role in elder people’s lives, which could prevent disability consequences such as falls, fall-related injuries, hospitalizations, and even mortality [21]. In this regard, our results show that 8 weeks of TST could improve functional tests such as gait speed, chair stand time, and time up and go (TUG) in sarcopenic elderlies. In line with this, previous studies reported improvements in older subjects’ muscle strength and functional capacity using TRX Suspension Training [52]. These studies have compared the effect of TST versus RT programs in older adults and the results showed similar muscle mass, strength, and functional performance among these two exercises [52]. On the other hand, it has been demonstrated that core muscles are activated more in TST exercises in comparison to similar RT exercises on the ground and TST had a significant increase in activation of at least one muscle group [53]. Considering the mentioned benefits of TST on skeletal muscle and functional performance, and the fact that it can be done in a small space like a home with affordable equipment, make it an ideal home-based training program, especially for older adults.

Like many studies, the current study is not without its limitations. Two potential limitations are identified in this study. Firstly, due to the implementation of the study during the COVID-19 period, the sample size is relatively small as individuals were reluctant to participate in sports environments with close contact. Secondly, the study did not have complete control over the subjects’ nutrition, which could potentially impact the results.

## Conclusion

The present study showed that TRX suspension Training could lead to improving functional abilities and biological markers involved in sarcopenia. After 8 weeks of TST, serum levels of Myostatin were significantly decreased and Follistatin, GDF-15, and CAF were significantly increased. However, it couldn’t significantly change ASMM and body composition in older men with sarcopenia. In addition, 8 weeks of TST improved the physical performance of the subjects as functional tests (Chair stand, TUG, and Gait speed) and handgrip strength. Therefore, our results demonstrated that the TRX Suspension Training is an effective protocol to improve the levels of muscle growth factors and functional abilities in the sarcopenic elderly. This training protocol presents itself as a suitable exercise regimen for this specific group of individuals, as it can be conveniently conducted as a home-based exercise. Moreover, this method is cost-efficient and portable, further enhancing its practicality.

It would be interesting for future research to investigate the effects of combining resistance training with dancing as aerobic training on the sarcopenic elderly [54]. Additionally, exploring the variances in changing CAF levels in response to exercise, along with elucidating the underlying mechanisms and pathways through which GDF-15 influences skeletal muscle mass, represent two other areas for further study. Electromyographical investigations alongside assessing CAF blood levels could clarify the role of exercise training on the neuromuscular junction in sarcopenic elderlies.

## Data Availability

The datasets used and/or analyzed during the current study are available from the corresponding author upon reasonable request.

## Declarations

### Authors’ information

SR: A researcher who recently received his Master’s degree in Exercise Physiology from Allameh Tabaaba’i University. His research focuses on studying mechanisms related to skeletal muscle mass and function and how exercise training, nutrition and other stimuli could affect them.

RE: RE has a Ph.D. in exercise physiology and He is an associate professor of physical education and sport science college of Allameh Tabataba’i University. His research interest is in neuromuscular adaptation to exercise training and he is the chief of a health center at Allameh Tabataba’i University.

BT: BT has a Ph.D. in exercise physiology and He is a professor of physical education and sport science college of Allameh Tabataba’i University. His research is on exercise nutrition, exercise immunology, and investigating the effect of exercise at the cellular and molecular levels.

I confirm that all authors have approved the manuscript for submission.

The content of the manuscript has not been published, or submitted for publication elsewhere.
